# Production of Hesperetin Glycosides by *Xanthomonas campestris* and Cyclodextrin Glucanotransferase and Their Anti-allergic Activities

**DOI:** 10.3390/nu2020171

**Published:** 2010-02-09

**Authors:** Kei Shimoda, Hiroki Hamada

**Affiliations:** 1 Department of Chemistry, Faculty of Medicine, Oita University, 1-1 Hasama-machi, Oita 879-5593, Japan; 2 Department of Life Science, Okayama University of Science, 1-1 Ridai-cho, Kita-ku, Okayama 700-0005, Japan

**Keywords:** Hesperetin, *Xanthomonas campestris*, cyclodextrin glucanotransferase, glycoside, anti-allergic activity

## Abstract

The production of hesperetin glycosides was investigated using glycosylation with *Xanthomonas campestris* and cyclodextrin glucanotransferase (CGTase). *X. campestris* glucosylated hesperetin to its 3'-, 5-, and 7-*O*-glucosides, and CGTase converted hesperetin glucosides into the corresponding maltosides. The resulting 7-*O*-glucoside and 7-*O*-maltoside of hesperetin showed inhibitory effects on IgE antibody production and on O_2_^-^ generation from rat neutrophils.

## 1. Introduction

Vitamin P is the term used for bioactive flavonoids. Hesperetin (5,7,3'-trihydroxyl-4'-methoxyl-flavanone), which occurs in *Citrus* species, tomatoes, and apples, is a kind of vitamin P and has been well documented for its medicinal properties as an important Chinese traditional medicine [1,2]. Hesperetin has been studied for its effects on the blood-brain barrier, signal transduction pathway, and certain kinds of cancer [3,4,5,6]. However, hesperetin barely dissolves in aqueous solution, and this low water-solubility limits its further pharmacological exploitation.

Glycosylation is considered to be an important method for the conversion of water-insoluble and unstable organic compounds into the corresponding water-soluble and chemically stable derivatives. Recently, Mizukami *et al.* reported that glucosyl conjugation was far more effective than cyclodextrin complexation at enhancing the water solubility of hydrophobic compounds such as curcumin [7]. Hesperidin is a hesperetin glycoside, *i.e.*, a 7-*O*-β-rutinoside of hesperetin. The values of the partition coefficient (logP) of hesperetin and hesperidin are 2.6 and 1.1, respectively [8]. Hesperidin is widely used as vitamin P2, which has biologically beneficial effects such as anticancer, antimutagenic, anticarcinogenic, and cholesterol-lowering activities [9,10,11]. Furthermore, the glycosides of hesperidin have been utilized in food industries as food additives (Hesperidin S (Hayashibara Biochemical Laboratories Ltd.)). Therefore, the glycosides of hesperetin are important from a physiological point of view. One-step biocatalytic glycosylation is useful for synthesis of glycosides rather than chemical glycosylation that requires tedious protection-deprotection procedures [7]. Herein we report the glycosylation of hesperetin into the corresponding glucosides and maltosides by *Xanthomonas campestris* and cyclodextrin glucanotransferase (CGTase), which has been used for production of Hesperidin S. We also report the inhibitory effects of hesperetin glycosides on IgE antibody formation and on O_2_^-^ generation from rat neutrophils. Hesperetin glycosides synthesized here may be useful food ingredients which have potent anti-allergic activities.

## 2. Results and Discussion

### 2.1. Production of Hesperetin α-glycosides

**Figure 1:**
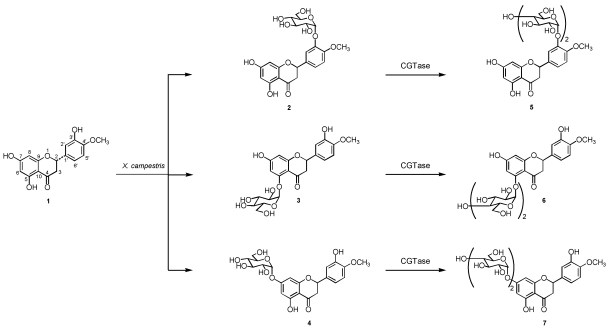
Synthetic route of α-glucosides and α-maltosides of hesperetin-------sequential biocatalytic glycosylation of hesperetin by *X. campestris* and CGTase.

The structure of hesperetin and the six newly synthesized vitamin P compounds are shown in Figure 1. The glucosylation of hesperetin (**1**) was investigated using the lyophilized cells of *X. campestris*. Incubation of lyophilized *X. campestris *cells with hesperetin (**1**) and a sugar donor, *i.e.*, maltose, for five days gave three new compounds, 3'-, 5-, and 7-*O*-α-glucosides of hesperetin (**2**-**4**), which were detected in HEPES-NaOH buffer (10 mM) by HPLC. The glucoside products **2**-**4** were isolated from *n*-butanol extracts of the buffer by preparative HPLC (**2**, 12% yield; **3**, 10%; **4**, 15%). The compounds **2**-**4** were individually glycosylated by CGTase to give three new compounds **5**-**7** in 49, 62, and 50% yield, respectively. The chemical structures of products **5**-**7** were determined as hesperetin 3'-*O*-α-maltoside, hesperetin 5-*O*-α-maltoside, and hesperetin 7-*O*-α-maltoside, which have not been identified before, by HRFABMS, ^1^H and ^13^C NMR, H-H COSY, C-H COSY, and HMBC spectra.

The HRFABMS spectra of **5**-**7** included pseudomolecular ion [M+Na]^+^ peaks at 649.1751 for **5**, 649.1755 for **6**, and 649.1750 for **7** (calculated for C_28_H_34_O_16_Na, 649.1745), indicating that each product consisted of one substrate and two hexoses. The sugar components in these products were determined to be glucose on the basis of the chemical shifts of their carbon signals. The ^1^H NMR spectra showed two proton signals at *δ* 5.01 (1H, *d*, *J* = 3.6 Hz) and 5.06 (1H, *d*, *J* = 3.2 Hz) for **5**, *δ* 5.03 (1H, *d*, *J* = 3.0 Hz) and 5.40 (1H, *d*, *J* = 3.2 Hz) for **6**, and *δ* 5.05 (1H, *d*, *J* = 3.2 Hz) and 5.17 (1H, *d*, *J* = 3.0 Hz) for **7**, indicating that the glucoside linkage in these three compounds had α-orientations. The HMBC spectra of **5**-**7** included correlations between the proton signal at *δ* 5.01 (H-1''') and the carbon signal at *δ* 80.7 (C-4'') and between the proton signal at *δ* 5.06 (H-1'') and the carbon signal at *δ* 146.2 (C-3') for **5**, between the proton signal at *δ* 5.03 (H-1''') and the carbon signal at *δ* 80.5 (C-4'') and between the proton signal at *δ* 5.40 (H-1'') and the carbon signal at *δ* 165.1 (C-5) for **6**, and between the proton signal at *δ* 5.05 (H-1''') and the carbon signal at *δ* 80.5 (C-4'') and between the proton signal at *δ* 5.17 (H-1'') and the carbon signal at *δ* 166.9 (C-7) for **7**. These data indicated that **5**-**7** were α-maltosyl analogues of **1**, the sugar moiety of which attached at their 3'-position (**5**), 5-position (**6**), and 7-position (**7**). Thus, products **5**-**7** were identified as hesperetin 3'-*O*-α-maltoside (**5**), hesperetin 5-*O*-α-maltoside (**6**), and hesperetin 7-*O*-α-maltoside (**7**), respectively. The substrate hesperetin used for this experiment was a chiral compound, the stereochemistry of which was 2*S* [12,13,14,15,16,17,18,19] (Sigma-Aldrich Co.). On the other hand, it has been reported that hesperidin epimerizes in C2 to form the 2*S* and 2*R* epimers [20]. The absolute configuration at C-2 of the aglycone of hesperetin α-glycosides synthesized here is not clarified.

It has been reported that kojic acid α-glucoside was synthesized by lyophilized *X. campestris* in the presence of maltose as a sugar donor [21]. This report led us to produce new vitamin P compounds, that is, hesperetin α-glycosides, by lyophilized *X. campestris*. Six new vitamin P compounds, *i.e.*, hesperetin α-glucosides and hesperetin α-maltosides, were successfully synthesized through sequential biocatalytic α-glycosylation by *X. campestris* and CGTase. Earlier, some kinds of hesperetin β-glycosides, such as β-glucosides, β-diglucosides, and β-rutinoside, had been isolated from fruits and flowers of *Citrus* species [22,23,24]. On the other hand, it has been reported that cultured cells of *Citrus paradisi* glycosylated hesperetin at its 7-position to give 7-*O*-β-glucoside and 7-*O*-β-rutinoside [25,26]. Compared to the β-glucosylation with *C. paradisi*, *X. campestris* showed high potential for the α-glucosylation at each phenolic hydroxyl groups of hesperetin to give three α-glucosides. This is the first report on the synthesis of hesperetin α-glycosides.

### 2.2. Anti-allergic Activity of Hesperetin α-glycosides

The effects of hesperetin α-glucosides **2**-**4 **and hesperetin α-maltosides **5**-**7** on IgE antibody formation were investigated by *in vivo* bioassay using ovalbumin as an antigen [27]. The average rat plasma IgE level after treatment of ovalbumin with or without test compounds was examined and the results are summarized in Table 1. Hesperetin 7-*O*-α-glucoside (**4**) and 7-*O*-α-maltoside (**7**) showed inhibitory action on IgE antibody generation. On the other hand, 3'-*O*-α-glycosides **2** and **5** and 5-*O*-α-glycosides **3** and **6** did not inhibit IgE antibody generation.

There is evidence that superoxide anion (O_2_^-^), which is generated primarily through the activation of the plasma membrane-bound NADPH-oxidase system, is responsible for allergic reactions, and anti-allergic drugs such as mequitazine have been reported to inhibit O_2_^-^ generation [28].

**Table 1:** Suppressive action of hesperetin α-glycosides **2**-**7** on IgE antibody formation.

Compound	IgE level^a^
None	393.1 ± 188.7
**2**	356.6 ± 186.9
**3**	338.3 ± 163.0
**4**	155.4 ± 67.2*
**5**	365.7 ± 126.7
**6**	374.9 ± 169.1
**7**	164.6 ± 57.8*

^a^The results were expressed as average plasma IgE level of seven rats administered a total of 10 mg/kg of each test compound. Data are presented as mean ± SE. Asterisk indicates significant differences from controls (*P < 0.05).

**Table 2:** Effects of hesperetinα-glycosides **2**-**7** on O_2_^-^ generation from rat neutrophils.

Compound	%Inhibition^ a^
**2**	12
**3**	17
**4**	59
**5**	5
**6**	7
**7**	46
Mequitazine	62

^a^The percentage reduction of the O_2_^-^ generation was calculated as follows: %Inhibition = [(*P*_c_- *P*_t_)/*P*_c_]x100, where *P*_t_ and *P*_c_ are the photon count of sample solutions with and without the test compound.

The effects of hesperetin α-glucosides **2**-**4** and hesperetin α-maltosides **5**-**7** on O_2_^-^ generation from rat neutrophils using cypridina luciferin analog-dependent chemiluminescence as a probe for O_2_^-^ generation were examined according to the previously reported methods [29]. The results are expressed in terms of the percentage reduction of the O_2_^-^ generation. Percent inhibition was calculated as follows: %Inhibition = [(*P*_c_- *P*_t_)/*P*_c_]x100, where *P*_t_ and *P*_c_ are the photon count of sample solutions with and without the test compound. As a result, inhibitory effects of hesperetin 7-*O*-α-glycosides **4 **and **7** and an authentic anti-allergic drug on fMLP-induced O_2_^-^ generation from rat neutrophils are as follows: **4**, 59%; **7**, 46%; mequitazine, 62% (Table 2). Particularly, hesperetin 7-*O*-α-glucoside (**4**) exhibited significant inhibitory activity for O_2_^-^ generation, to the same level as mequitazine. This is the first time that hesperetin α-glycosides have been shown to have a strong inhibitory effect on O_2_^-^ generation. Hesperetin 3'-*O*-α-glycosides **2** and **5** and hesperetin 5-*O*-α-glycosides **3** and **6** showed no inhibitory action on O_2_^-^ generation. Previously, we reported that 7-*O*-β-glycosides of polyphenols, *i.e.*, genistein and quercetin, showed anti-allergic activities, whereas polyphenol glycosides, where the is attached at other phenolic hydroxyl groups, exhibited no anti-allergic actions [29]. These findings suggest that α-glycosides at C-7 of hesperetin did not attenuate the anti-allergic activity of the aglycone, *i.e.*, hesperetin, and that phenolic hydroxyl groups at 3'- and 5-positions of hesperetin might be necessary for hesperetin and its glycosides to act as anti-allergic formulations. Recently, it has been reported that hesperetin is more bioactive than its glycoside hesperidin, and efforts to formulate hesperetin are in progress to improve absorption and its disposition [30,31,32,33]. The hesperetin α-glycosides might act as pro-drugs that release hesperetin by hydrolysis in the living body.

Recently, some β-glycosides of tocopherol (vitamin E) have been reported to act as potential anti-allergic agents [34]. In addition, the β-glycosides of vitamin E showed inhibitory effects on O_2_^-^ generation from rat neutrophils. It was postulated that inhibition of O_2_^-^ generation by vitamin E β-glycosides was responsible for their anti-allergic activity [28,34]. Studies on the mechanism by which the hesperetin α-glycosides synthesized here act as anti-allergic compounds are now in progress.

## 3. Experimental Section

### 3.1. General

Substrate, hesperetin, was purchased from Sigma-Aldrich Co. CGTase was purchased from Amano Pharmaceutical Co. Ltd. The NMR spectra were recorded in CD_3_OD using a Varian XL-400 spectrometer. The chemical shifts were expressed in δ (ppm) referring to tetramethylsilane. The HRFABMS spectra were measured using a JEOL MStation JMS-700 spectrometer. HPLC was carried out on a YMC-Pack R&D ODS column (150 x 30 mm) [solvent: CH_3_CN:H_2_O (3:17, v/v); detection: UV (280 nm); flow rate: 1.0 mL/min]. 

### 3.2. Bacterial Strain and Culture Conditions

*X. campestris *was a gift from the Department of Chemistry of Okayama University of Science. Culture medium used for growth of *X. campestris* had the following composition (in grams per liter): 5 g of maltose, 3 g of peptone, 0.4 g of yeast extract, 0.2 g of MgSO_4_. The cells were grown in the culture medium with continuous shaking on a rotary shaker (120 rpm) at 30°C.

### 3.3. Production of Hesperetin α-glucosides by X. Campestris

The cultures of *X. campestris* were grown in 500 mL conical flasks containing 200 mL of culture medium at 30 °C. Prior to use for the experiments, the cells were harvested by centrifugation at 8,000 g for 15 min and were lyophilized. Hesperetin α-glucosides were prepared as follows. A total of 2 mmol of hesperetin was added to ten 300-ml conical flasks (0.2 mmol/flask), each of which contained 5 g of lyophilized *X. campestris* cells and 1 mmol of maltose in 100 ml of 10 mM HEPES-NaOH buffer (pH 7.5). The mixture was incubated continuous shaking on a rotary shaker (120 rpm) for 5 days at 30 °C. The reaction mixture was centrifuged at 8,000 g for 15 min and the supernatant was extracted with *n*-butanol. The butanol fraction was purified by preparative HPLC on YMC-Pack R&D ODS column to give hesperetin α-glucosides.

### 3.4. Production of Hesperetin α-maltosides by CGTase

To a solution containing 0.1 mmol of hesperetin α-glucoside and 5 g of starch in 25 mM of sodium phosphate buffer (pH 7.0) was added 100 U of CGTase. The reaction mixture was stirred at 40 °C for 24 h, and then the mixture was centrifuged at 3,000 g for 10 min. The supernatant was subjected on to a Sephadex G-25 column equilibrated with water to remove CGTase. The fractions containing glycosides were purified by preparative HPLC on YMC-Pack R&D ODS column to give hesperetin α-maltosides.

Spectral data of selected new compounds are as follows.

Hesperetin 3'-*O*-α-maltoside (**5**): FABMS *m/z*: 649.1751 [M+Na]^+^; ^1^H NMR (400 MHz, DMSO-*d*_6_, δ in ppm): *δ* 2.77 (1H, *dd*, *J* = 17.8, 2.8 Hz, H-3a), 3.08-3.88 (12H, m, H-2'', 2''', 3'', 3''', 4'', 4''', 5'', 5''', 6'', 6'''), 3.33 (1H, *dd*, *J* = 17.8, 12.8 Hz, H-3b), 3.78 (3H, *s*, OCH_3_), 5.01 (1H, *d*, *J* = 3.6 Hz, H-1'''), 5.06 (1H, *d*, *J* = 3.2 Hz, H-1''), 5.50 (1H, *dd*, *J* = 12.6, 2.8 Hz, H-2), 5.89 (1H, *d*, *J* = 2.8 Hz, H-6), 5.90 (1H, *d*, *J* = 2.8 Hz, H-8), 7.03 (1H, *dd*, *J* = 8.4, 2.0 Hz, H-6'), 7.08 (1H, *d*, *J* = 8.4 Hz, H-5'), 7.25 (1H, *d*, *J* = 2.0 Hz, H-2'); ^13^C NMR (100 MHz, DMSO-*d*_6_, δ in ppm): δ 43.3 (C-3), 55.5 (OCH_3_), 60.5 (C-6'''), 60.7 (C-6''), 69.5 (C-4'''), 72.2 (C-5''), 72.4 (C-2''), 73.5 (C-2''', C-3''), 73.9 (C-3''', C-5'''), 78.0 (C-2), 80.7 (C-4''), 94.7 (C-8), 95.7 (C-6), 98.0 (C-1''), 100.5 (C-1'''), 101.4 (C-10), 111.5 (C-5'), 113.6 (C-2'), 120.5 (C-6'), 130.2 (C-1'), 146.2 (C-3'), 149.0 (C-4'), 162.4 (C-9), 163.2 (C-5), 166.3 (C-7), 196.9 (C-4).

Hesperetin 5-*O*-α-maltoside (**6**): FABMS *m/z*: 649.1755 [M+Na]^+^; ^1^H NMR (DMSO-*d*_6_): δ 2.76 (1H, *dd*, *J* = 17.8, 2.8 Hz, H-3a), 3.05-3.89 (12H, m, H-2'', 2''', 3'', 3''', 4'', 4''', 5'', 5''', 6'', 6'''), 3.33 (1H, *dd*, *J* = 17.8, 12.8 Hz, H-3b), 3.78 (3H, *s*, OCH_3_), 5.03 (1H, *d*, *J* = 3.0 Hz, H-1'''), 5.40 (1H, *d*, *J* = 3.2 Hz, H-1''), 5.51 (1H, *dd*, *J* = 12.6, 2.8 Hz, H-2), 6.08 (1H, *d*, *J* = 2.8 Hz, H-6), 6.12 (1H, *d*, *J* = 2.8 Hz, H-8), 6.90 (1H, *dd*, *J* = 8.4, 2.0 Hz, H-6'), 7.00 (2H, *m*, H-2', 5'); ^13^C NMR (DMSO-*d*_6_): δ 43.0 (C-3), 55.5 (OCH_3_), 60.3 (C-6'''), 60.6 (C-6''), 69.5 (C-4'''), 72.2 (C-5''), 72.5 (C-2''), 73.5 (C-2''', C-3''), 74.1 (C-3''', C-5'''), 78.0 (C-2), 80.5 (C-4''), 93.7 (C-8), 94.8 (C-6), 99.7 (C-1''), 100.6 (C-1'''), 103.5 (C-10), 111.7 (C-5'), 113.8 (C-2'), 117.7 (C-6'), 130.8 (C-1'), 146.0 (C-3'), 147.7 (C-4'), 162.5 (C-9), 165.1 (C-5), 165.0 (C-7), 198.9 (C-4).

Hesperetin 7-*O*-α-maltoside (**7**): FABMS *m/z*: 649.1750 [M+Na]^+^; ^1^H NMR (DMSO-*d*_6_): δ 2.76 (1H, *dd*, *J* = 17.8, 2.8 Hz, H-3a), 3.07-3.83 (12H, m, H-2'', 2''', 3'', 3''', 4'', 4''', 5'', 5''', 6'', 6'''), 3.32 (1H, *dd*, *J* = 17.8, 12.8 Hz, H-3b), 3.78 (3H, *s*, OCH_3_), 5.05 (1H, *d*, *J* = 3.2 Hz, H-1'''), 5.17 (1H, *d*, *J* = 3.0 Hz, H-1''), 5.49 (1H, *dd*, *J* = 12.6, 2.8 Hz, H-2), 6.16 (1H, *d*, *J* = 2.8 Hz, H-6), 6.22 (1H, *d*, *J* = 2.8 Hz, H-8), 6.88 (1H, *dd*, *J* = 8.4, 2.0 Hz, H-6'), 6.92 (2H, *m*, H-2', 5'); ^13^C NMR (DMSO-*d*_6_): δ 42.2 (C-3), 55.5 (OCH_3_), 60.4 (C-6'''), 60.6 (C-6''), 69.5 (C-4'''), 72.2 (C-5''), 72.5 (C-2''), 73.8 (C-2''', C-3''), 74.1 (C-3''', C-5'''), 78.2 (C-2), 80.5 (C-4''), 95.4 (C-6), 95.9 (C-8), 100.1 (C-1''), 100.7 (C-1'''), 103.2 (C-10), 111.5 (C-5'), 113.9 (C-2'), 117.6 (C-6'), 130.7 (C-1'), 146.0 (C-3'), 147.8 (C-4'), 162.2 (C-9), 162.7 (C-5), 166.9 (C-7), 196.7 (C-4).

### 3.5. Suppressive Action on IgE Antibody Formation

The inhibitory action of hesperetin α-glycosides on IgE antibody formation was examined as follows. Ovalbumin was used as the antigen (1 mg/rat), and Al(OH)_3_ and pertussis vaccine were used as the adjuvants (20 mg and 0.6 mL/rat, respectively). Sensitization was made by injecting a mixture (0.6 mL) of the antigen and the adjuvant into the paws of each rat (male, ca. 200 g). Paw edema was measured 24 h after injection and the treated rats were divided in groups with an equal average swelling volume. Each sample was dissolved in physiological saline containing 10% Nikkol and the solution was injected daily into the rat for 11 days starting on the day of grouping. The amount of IgE was measured by the passive cutaneous anaphylaxis method on the 15th day [27]. The results were expressed as average of plasma IgE level of seven rats administered a total of 10 mg/kg of each test compound.

### 3.6. Inhibitory Action on O2- Generation from Rat Neutrophils

Effects of hesperetin α-glycosides on O_2_^-^ generation from rat neutrophils were examined as follows. Male Wistar rats, each weighing 250 to 300 g, were used. Under ether anesthesia, whole blood was collected from the carotid artery and diluted twice with Hanks' balanced salt solution (HBSS) (pH 7.4). Neutrophils were purified by Percoll density gradient centrifugation. O_2_^-^ generation from rat neutrophils was measured by the cypridina luciferin analog-dependent chemiluminescence method [28]. An authentic antiallergic agent, mequitazine, was used as a positive control. Neutrophil suspensions (10^6^ cells/mL) were incubated for 3 min in HBSS containing 0.4 mM of cypridina luciferin analog and 50 μM of hesperetin α-glycoside or authentic antiallergic agent at 37 °C in the dark. Five seconds later, *N*-formyl-Met-Leu-Phe (fMLP) (2.5 μM) was added into the assay mixture. Cypridina luciferin analog-dependent chemiluminescence was monitored with a Lumicounter ATP-237 (Advantec Co. Ltd.). The results are expressed in terms of the percentage reduction of the O_2_^-^ generation from rat neutrophils at 5 min after the administration of fMLP by test compounds.

## 4. Conclusions

Hesperetin α-glycosides, *i.e.*, α-glucosides and α-maltosides, were successfully synthesized through sequential biocatalytic glycosylation by *X. campestris* and CGTase for the first time. It should be emphasized that new vitamin P glycosides, *i.e.*, hesperetin α-maltosides, which are glycosylated at each phenolic hydroxyl group of hesperetin, can be prepared by the present two-step glycosylation system. Hesperetin 7-*O*-α-glucoside and hesperetin 7-*O*-α-maltoside showed suppressive action on IgE antibody formation and exhibited inhibitory effects on O_2_^-^ generation from rat neutrophils. These new vitamin P compounds would be useful food ingredients with potent anti-allergic activities. Further studies on the physiological activity of hesperetin α-glycosides, such as cytotoxicity and anticancer activity, are now in progress.
